# Round about the Asylums. III

**Published:** 1888-03-24

**Authors:** 


### Round About the Asylums.—III.

By a Medical Superintendent.

### EXETER BOROUGH ASYLUM.

This is the most recently-built asylum in England, and was opened in the autumn of 1886. It has thus just completed its first year of existence. On the last day of December, 1887, it contained 174 patients—169 being paupers and five private cases. The visitors remark that "the chance of recovery is fearfully lessened, if not entirely destroyed, unless the patient is placed under treatment in the early stage of the disorder." This is not a new saying, but it is unquestionably a true one, and therefore it can never be wrong to repeat and emphasize it in the hope that it may imprint itself on the cerebral palimpsest of those responsible for sending the insane to asylums; or should we say of those responsible for keeping them at home too long before sending them? The maintenance cost is 12*s*. per head per week. This may seem rather high, but, as the visitors explain, a new asylum is more expensive to work than an old one; and, taking into consideration this fact and also that there are only 174 patients, we do not think it excessive, especially if the officers and attendants are sufficiently paid. Dr. Rutherford gives a long description of the asylum through which we need not follow him, further than to say that the electric light is used, that a Turkish bath is provided, and that the superintendent's residence is a detached building. All these are good things in their way, and every superintendent will envy him the last-mentioned at least. The recoveries were 26 per cent. on the admissions, and the deaths 9·6 on the average number resident. It is stated that the assistant medical officer was appointed in January, 1886, although the asylum was not opened until September, 1886. Is this a misprint? The Commissioners say that the slippery condition of the cement floors and stairs is a great defect, and they reasonably predict the occurrence of accidents unless this defect is rectified. How comes it that this blemish exists in so new an asylum? The Commissioners remark that the cash-books are well kept, and that the medical officers are earnest in their efforts to promote the patients' welfare. A ground plan of the asylum accompanies the report, from which we learn that the building is constructed on the old gallery plan, and that several wards can only be reached by passing through other wards, principles of construction which we had thought were as extinct as the dodo.

### ENNIS DISTRICT ASYLUM FOR CLARE COUNTY.

To the majority of English medical superintendents an Irish asylum is a sort of *terra incognita*. He has heard of them, and possibly read some of their reports, but has only vague ideas of what they are really like. The Clare Asylum contains 350 patients. The numbers increased by 22 during the year. The Lunacy Inspector suggests the enlargement of the asylum by 80 beds, and in the meantime some arrangements have been made whereby an additional 30 beds have been obtained at a small cost. The weekly cost of maintenance is a little under 9*s*. It would seem that even an extra attendant cannot be appointed here without the consent of the Privy Council, as mention is made of such an appointment. Two attendants received pensions, and it is pleasing to note that these were the only changes among the staff of attendants during the year 1887. The recoveries were 32·8 per cent. and the deaths 4·9 per cent., both calculated in the manner common in English asylums. No annual report is presented by the committee, and the auditing of the books is apparently done by an auditor and not by the committee, as required by the English Lunacy Acts. With the condition of the asylum the Lunacy Inspector expresses his "unreserved approbation." During the year the asylum lost the services by death of the late medical superintendent, Dr. Dixon, and Dr. Gelston now reigns in his stead.

### CUMBERLAND AND WESTMORELAND ASYLUM.

In this institution, which is familiarly known as the Garlands Asylum, there are 575 patients, 47 of these being out-county and private patients. The weekly rate is 8*s*. 2*d*. for patients chargeable to their own unions, 14*s*. for out counties, and 17*s*. 6*d*. for private cases. The visitors' report is extremely short. It says nothing about the staff—a curious omission, as this is one of the points which the Lunacy Acts require the committee to report on to Quarter Sessions. During the year four patients were restrained for periods varying from twenty-four hours to seventeen days for surgical reasons, and eleven patients were secluded occasionally. Occupation of the patients seems carefully attended to. It is stated that 80 per cent. of the men and 60 per cent of the women are employed. The recovery-rate stands at 40·4 per cent. on the admissions, and the death-rate at 7·7 per cent. on the average number resident. The general history shows that the Cumberland and Westmoreland patients increased by twenty-one; that there was no serious accident; that fourteen patients escaped; and that (what is often experienced) the changes among the female staff were too numerous. Dr. Campbell says: "The nature of asylum work is undoubtedly trying and irksome; but either the inducements offered in this asylum are insufficient, or else the love of novelty and change which exists in the female mind overcomes present benefits as well as the prospect of future reward for long and good service." It would be interesting to know whether Dr. Campbell engages nurses from other asylums. If so, it would account for some of the changes. Incorporated with the superintendent's report are some sensible remarks on lunacy legislation, the most important of which is that power should be obtained to transfer Irish and Scotch lunatics to their own countries. The necessity for this has already been pointed out in the medical and lay papers; but there would seem to be some strange and unaccountable objection to the insertion of such a greatly wanted clause in any Lunacy Bill, although the necessity for it cannot be denied by anyone. In the asylum under notice foreigners are kept at the annual expense of over £500 a-year to the already overburdened English ratepayers; and in Lancashire £500 is a mere fleabite compared to the sum which has to be expended on those with whom the ratepayers ought to have nothing to do. The new Lunacy Bill will be under discussion immediately. English M.P.'s should decline to vote for any Bill which does not contain the clause! It is now or never!

NORWICH CITY ASYLUM.

At the end of the year there were 230 patients in the asylum, only one being of the private class. There were 73 admissions during the year, and the discharges by recovery and death almost exactly balanced the admissions, there being 229 patients under treatment at the beginning of 1887, and, as already stated, 230 at the close. The recoveries were nearly 31 per cent. on the admissions, and the deaths were 12·98 per cent. on the average number resident. Dr. Harris explains this high death-rate as follows: "During the year several patients have been admitted in a hopeless or dying state. Surely it would have been kinder to have allowed them to breathe their last in their own homes, though the trifling expense of special nursing had been incurred." There is something in this remark, still it should not be forgotten that a public asylum is intended for all sorts of cases, and it is doubtful whether persons afflicted with insanity plus some serious organic disease, could be properly dealt with in a cottage or in the wards of a workhouse. Dr. Harris would, however, seem to have had many extreme cases, as several of the admissions died within a few weeks, some even within a few days, of their entering the asylum. The weekly rate of maintenance is 8*s.* 9*d.*, which is creditable enough provided the salaries and wages are sufficiently high. No detailed statement of them is given; but we learn that the aggregate amounts to barely 2*s.* 5¾*d.* per week per head; and we doubt whether it is possible to do it for this sum in a liberal manner where there are only 230 patients. This is one of the very few county or borough asylums having no assistant medical officer, but we notice that the Commissioners advise the appointment being made.

